# NAD^+^-boosting molecules suppress mast cell degranulation and anaphylactic responses in mice

**DOI:** 10.7150/thno.69684

**Published:** 2022-04-11

**Authors:** Hyun-Woo Kim, Ga-Hee Ryoo, Hyun-Young Jang, So-Young Rah, Dong Hyun Lee, Do-Kyun Kim, Eun Ju Bae, Byung-Hyun Park

**Affiliations:** 1 Korea Zoonosis Research Institute, Chonbuk National University, Iksan 54596, Republic of Korea.; 2 Department of Biochemistry and Molecular Biology, Chonbuk National University Medical School, Jeonju 54896, Republic of Korea.; 3 Department of Obstetrics and Gynecology, Chonbuk National University Medical School, Jeonju 54896, Republic of Korea.; 4 College of Pharmacy, Chonbuk National University, Jeonju 54896, Republic of Korea.

**Keywords:** Sirt6, mast cell, anaphylaxis, NMN, NR

## Abstract

Nicotinamide adenine dinucleotide (NAD^+^) acts as a cofactor for multiple biological processes. While previous research has revealed that the NAD^+^ declines associated with aging contributes to an impairment of immune cells, its role in mast cell function, especially in response to an anaphylactic condition, has remained unexplored. We tested whether the restoration of cellular NAD^+^ concentration by the supplementation of NAD^+^ boosting molecules prevented mast cell degranulation and anaphylactic responses.

**Methods:** Bone marrow derived mast cells (BMMCs) and human cord blood derived mast cells were treated with NAD^+^ precursors nicotinamide mononucleotide (NMN) and nicotinamide riboside (NR), and FcεRI downstream signaling was assessed. Animal models of passive systemic anaphylaxis (PSA) and passive cutaneous anaphylaxis (PCA) were used to investigate the effects of NAD^+^ precursors in the anaphylactic responses of mice.

**Results:** Treatment of murine BMMCs and human cord blood derived mast cells with NAD^+^ precursors repressed intracellular signaling downstream of FcεRI, as well as the release of inflammatory cytokines and lipid mediators. The intraperitoneal administration of NMN or NR also markedly attenuated IgE-mediated anaphylactic responses in mouse models of PSA and PCA. These beneficial effects of NAD^+^ precursors, however, were attenuated in mast cell-specific *Sirt6* knockout mice, indicating a Sirt6 dependency for their action.

**Conclusion:** NAD^+^ precursors may serve as an effective therapeutic strategy that limits mast cell-mediated anaphylactic responses.

## Introduction

Anaphylaxis is an acute and life-threatening systemic response at the extreme end of the allergic continuum that can be triggered by foods, drugs, or chemical toxins. Risk factors for anaphylaxis include asthma history, old age, and comorbidities 1. Although the pathogenesis is not completely understood, IgE-mediated activation of mast cells is a known hallmark of the anaphylactic reaction 2. Mast cells are located in connective tissues throughout the body and express the high-affinity IgE receptor FcεRI on their surface. Crosslinking of IgE-bound FcεRI by polyvalent antigens triggers a series of intracellular signaling cascades that lead to a massive release of inflammatory mediators, including β-hexosaminidase, histamine, proteases, and cytokines 3. Despite extensive research, treatment for this fatal condition remains largely supportive with no specific effective therapies having been identified to date. General therapeutic modalities to prevent the allergic response such as avoidance of triggering allergen and administration of antihistamines may be partly effective. However, once an anaphylactic reaction has begun, injection with epinephrine, which is a standard first-line treatment is usually indicated 4.

Nicotinamide adenine dinucleotide (NAD^+^) functions as a coenzyme in cellular redox reactions. NAD^+^, once known only as an essential element in cellular metabolism, is today understood to be a key regulator of fundamental biological processes, such as DNA repair, cellular stress responses, and cell survival 5. In mammals, NAD^+^ is synthesized through either the *de novo* pathway from tryptophan, or the salvage pathway from nicotinamide mononucleotide (NMN) or nicotinamide riboside (NR) 6. Previous studies have shown that NAD^+^ is reduced in individuals with age-associated pathophysiologies, such as obesity and type 2 diabetes 7. Moreover, it was recently reported that both acute and chronic inflammation cause a reduction in NAD^+^, a result that links inflammation to age-related diseases 8. Consistently, supplementation with NMN or NR ameliorates metabolic, age-related, neurodegenerative, and inflammatory diseases 9. A leading candidate mechanism of NAD^+^ therapeutic activity involves the activation of sirtuins, which are NAD^+^-dependent histone and non-histone protein deacetylases. In cardiac muscle, for example, NMN supplementation restores cardiac function to near-normal levels in mouse models with Friedreich's ataxia cardiomyopathy through the upregulating of mitochondrial Sirt3 expression 10. NR supplementation also reduces organ injury and improves survival in septic mice through Sirt1 activation 11.

We recently determined that Sirt6 plays an essential role in regulating mast cell activation in response to allergen exposure 12. Despite numerous studies showing the benefits of NR and NMN supplementation in various disease models, experiments to determine the effects of NMN and NR on anaphylaxis have not been widely reported. In a 1974 study, Bekier et al. 13 demonstrated that nicotinamide, another key NAD^+^ intermediate, inhibited histamine release from rat peritoneal mast cells by compound 48/80, suggesting possible beneficial effects of NAD^+^ precursors on anaphylaxis. Thus, we sought to determine the efficacy of NMN or NR supplementation on anaphylactic responses and test the role of Sirt6 in mediating the effects of NAD^+^ precursors.

## Materials and Methods

### Animals

Six-week-old male C57BL/6J mice were purchased from Samtako Bio Korea (Osan, Korea). To generate mast cell-specific *Sirt6* KO mice, *Sirt6^flox/flox^* mice (Jackson Laboratory, Bar Harbor, ME, USA) were backcrossed for at least eight generations to the C57BL/6J background and crossed with *Cma1-Cre* (C57BL/6J background) mice (Jackson Laboratory). Mice were housed in a room at a temperature of 22 ± 2 °C and humidity of 55 ± 5%. All animal experiments were performed in accordance with the US National Institutes of Health's Guide for the Care and Use of Laboratory Animals (NIH Publication No. 85-23, revised 2011). The study protocol was approved by the Institutional Animal Care and Use Committee of Chonbuk National University (Permit No: CBNU 2021-010).

### Passive systemic anaphylaxis (PSA) and passive cutaneous anaphylaxis (PCA)

Seven-week-old male mice were sensitized by intravenous injection with 10 µg of anti-DNP IgE in 100 µL PBS. The following day, mice were intravenously challenged with 100 μg of DNP-HSA in 100 µL PBS. Body temperature was measured with a rectal thermometer (eDAQ, NSW, Australia) after challenge every ten min for 90 min. For PCA, seven-week-old male mice were sensitized by intradermal injection with 1 µg of anti-DNP-IgE in 20 μL PBS in the right ear and an equal volume of saline in the left ear. After 24 h, mice were challenged intravenously with DNP-HSA (100 μg in saline containing 1% Evans blue). Thirty minutes after the challenge, skin areas were photographed and the thickness of the ears was measured, after which the mice were euthanized. Evans blue dye was extracted by incubating the skin tissues in 700 μL of formamide for 24 h at 63 °C, after which absorbance was measured on a spectrophotometer at 620 nm. NMN (Sigma-Aldrich, St. Louis, MO, USA) and NR (MedKoo Biosciences, Morrisville, NC, USA) were dissolved in saline and administered intraperitoneally three times before IgE sensitization.

### Histology

Tissues were fixed with 10% formalin and embedded in paraffin. Fixed tissues were cut into 5 μm sections and deparaffinized. Sections were stained with hematoxylin-eosin (H&E) or toluidine blue for light microscopic examinations or mast cell infiltration (number of mast cells under 200× magnification field), respectively. The percentage of degranulated cells (number of degranulated mast cells per total number of mast cells under 200× magnification field) was analyzed using iSolution DT36 software (Carl Zeiss, Oberkochen, Germany) at the Center for University-wide Research Facilities (Chonbuk National University, Jeonju, Korea). Mast cell counts were performed by an independent observer.

### Toxicity study

NMN or NR was administered intraperitoneally (100 or 150 mg/kg/day, respectively) for three consecutive days. Plasma levels of hemoglobin (Hb), hematocrit (Hct), as well as the number of white blood cells (WBC) and platelets (PLT) were measured using an automated hematology analyzer (XN-2000, Sysmex Co., Kobe, Japan). Plasma levels of alanine aminotransferase (AST), aspartate aminotransferase (AST), blood urea nitrogen (BUN) and creatinine (Cr) were measured using an automated chemistry analyzer (ADVIA 2400, Siemens Healthcare Diagnostics Inc., Tarrytown, NY, USA).

### Bone marrow-derived mast cells (BMMCs)

Bone marrow cells were obtained by flushing bone marrow from the femurs and tibias of mice. Cells were cultured in BMMC media (RPMI 1640 media containing 2 mM L-glutamine, 10% FBS, 1 mM sodium pyruvate, 0.1 mM nonessential amino acids, 100 U/mL penicillin, 100 mg/mL streptomycin, 0.05 mM 2-mercaptoethanol, 25 mM HEPES, 10 ng/mL IL-3, and 10 ng/mL stem cell factor (SCF, PeproTech, Cranbury, NJ, USA). After 4-6 weeks in culture, BMMCs were stained to confirm the surface expression of FITC-anti-mFcεRI (MAR-1, eBioscience, San Diego, CA, USA) and PE-anti-mCD117 (2B8, BD Biosciences, San Jose, CA, USA). Cells with purity > 95% were used for subsequent experiments.

### Generation of human cord blood derived mast cells (hCBMCs)

Umbilical cord blood was obtained from normal vaginal and cesarean deliveries. Informed consent was received prior to the collection of cord blood and the study was approved by the Institutional Review Board of Chonbuk National University Hospital (Approval No. CBNUH: 2020-07-039-004). hCBMCs were generated as described previously 12. Briefly, mononuclear cells were isolated by layering heparin-treated cord blood onto a Ficoll-Paque solution (GE healthcare, Chicago, IL, USA). CD34^+^ progenitor cells were isolated by magnetic cell sorting kit (CD34 Microbead kit, Miltenyi Biotech, Auburn, CA, USA). For the first week, CD34^+^ progenitor cells were cultured in Iscove modified Dulbecco medium supplemented with 1% insulin-transferrin-selenium, 0.1% β-mercaptoethanol, 50 ng/mL rhIL-3, 50 ng/mL rhIL-6, and 50 ng/mL rhSCF (all recombinant cytokines from PeproTech). After 6 weeks, the cells were cultured in 50 ng/mL rhIL-6 and 50 ng/mL rhSCF. hCBMCs cultured for at least 15 weeks were used for experiments, and cell purity was greater than 98%. hCBMCs were identified by flow cytometric analysis. Cell viability was determined by trypan blue (0.4%) exclusion.

### Isolation of peritoneal mast cells

Eight-week-old male C57BL/6J mice were subjected to two sequential peritoneal lavages with 3 mL of ice-cold PBS. Cells were collected by centrifugation at 300 *g* for 3 min, washed with PBS, and resuspended in 100 μL of FACS buffer (PBS containing 2% BSA and 0.09% sodium azide). Cells were stained with FITC-conjugated FcεRI (134306, BioLegend, San Diego, CA, USA) and PE-conjugated CD117 (553355, BD Pharmingen, San Diego, CA, USA) for an hour in the dark. The FcεRI^+^CD117^+^ cells were subsequently sorted by FACSAria system (BD Biosciences) and used to measure intracellular NAD^+^ concentration.

### Flow cytometry

To determine the expression of tryptase, FcεRI and, CD117, hCBMCs were washed twice with PBS and blocked by CD16/32 monoclonal antibody (14-0161-86, Invitrogen, Carlsbad, CA, USA). The cell surface was stained with either FITC-conjugated anti-FcεRI (334607, BioLegend), PE-conjugated anti-CD117 (375305, BioLegend), or anti-tryptase (sc-33676, Santa Cruz Biotechnolgoy, Dallas, TX, USA) with FITC-conjugated rat anti-mouse IgG1 (11-4015-82, Invitrogen) in a FACS buffer. FITC-conjugated anti-FcεRI (134306, BioLegend) and PE-conjugated anti-CD117 (553355, BD Pharmingen) was used for BMMC surface staining, after which the cells were fixed with 4% paraformaldehyde solution and analyzed using an Accuri flow cytometer and FlowJo software (BD Biosciences). Dead cells were excluded through forward and side scatter (FSC/SSC) gating.

### Degranulation assay

BMMCs were incubated overnight at 37 °C with 100 ng/mL anti-DNP-IgE (SPE-7, Sigma-Aldrich) and then stimulated for 30 min at 37 °C with 100 ng/mL DNP-HSA antigen in 100 μL Tyrode's buffer. Control cells were treated with PBS. Culture supernatants were collected and cell pellets were lysed with 1% Triton X-100. To measure β-hexosaminidase activity, culture supernatant and cell lysates (30 μL) were incubated with 30 μL of substrate (1 mM p-nitrophenyl-N-acetyl-β-D-glucosaminide) for 60 min and then 0.1 M carbonate (250 μL) was added to stop the reaction. Absorbance of each compartment was measured at 405 nm. Percentage of β-hexosaminidase release was calculated as a percentage of total content, using the following formula: β-hexosaminidase release (%) = OD (supernatant)/[OD (supernatant)+OD (lysate)] × 100. For the hCBMCs degranulation assay, cells were incubated overnight with 100 ng/mL human myeloma IgE (Millipore, Temecula, CA, USA) and stimulated for 30 min with 100 ng/mL mouse-anti-human IgE (Invitrogen). Cells were then stained to confirm the surface expression of FITC-anti-LAMP-1 (H4A3) and PE-anti-hCD117 (104D2, Biolegend) by flow cytometric analysis.

### Enzyme linked immunosorbent assay (ELISA)

After sensitization and stimulation of BMMCs with 100 ng/mL anti-DNP-IgE and DNP-HSA, respectively, the supernatants were collected and the amounts of MCPT1, IL-6, TNF-α (eBioscience), LTC_4_, PGD_2_ (Cayman, Ann Arbor MI, USA), and histamine (Beckman Coulter, Brea, CA, USA) were calculated using ELISA kits consistent with manufacturer's instructions. At 100 min following the DNP-HSA challenge in the PSA model, we collected serum samples from the mice, and the levels of histamine, MCPT1, and IL-6 were determined using ELISA.

### Calcium (Ca^2+^) measurement

BMMCs (1 × 10^6^) were suspended in 1 mL culture medium and sensitized with 100 ng/mL anti-DNP-IgE overnight. Cells were washed twice with PBS and then loaded with 5 μM Fluo-4 AM (Molecular Probes, Eugene, OR, USA) for 30 min. Cells were subsequently washed again and further incubated in PBS for 30 min at room temperature. DNP-HSA (100 ng/mL) was then used to induce calcium flux, which was measured using a confocal microscope (Nikon, Tokyo, Japan) to monitor fluorescent emissions. Changes in intracellular calcium [Ca^2+^]_i_ was calculated using the following equation developed by Tsien *et al*. 14: [Ca^2+^]_i_ = *K*_d_ (*F* - *F*_min_)/(*F*_max_ - *F*), where *K*_d_ is 345 nM for Fluo-4 and *F* is the observed fluorescence level. Each tracing was calibrated for maximum intensity (*F*_max_) by the addition of 8 µM ionomycin, and for minimum intensity (*F_min_*) by the addition of 50 mM EGTA following each measurement.

### Confocal microscopy

Double-color immunofluorescence analysis was performed to determine colocalization in the BMMCs. Following treatment with anti-DNP-IgE and DNP-HSA, the BMMCs were washed with PBS and fixed with 2% formaldehyde for 30 min. After blocking with 5% normal goat serum, cells were incubated overnight at 4 °C with a combination of anti-FcɛRI (sc-393789, Santa Cruz Biotechnology, Dallas, TX, USA) and anti-PTPRC (ab10558, Abcam, Cambridge, UK), washed with PBS, and then incubated for 30 min with either fluorochrome-conjugated goat anti-rabbit IgG (A-11008, Invitrogen) or goat anti-mouse IgG2b (A-21145, Invitrogen). The cells were mounted and visualized using an LSM880 confocal laser scanning microscope (Carl Zeiss).

### Western blotting

Cell or tissue homogenates (20 μg) were separated by 10% SDS-PAGE and transferred to PVDF membranes. After blocking with 5% skim milk, blots were probed with primary antibodies against p-LAT (ab4476), Ac-H3K18 (ab1191), PLCγ (ab76155) (all from Abcam), LAT (sc-53550, Santa Cruz Biotechnology), Sirt6 (#12486), p-Syk (#2717), Syk (#13198), p-PLCγ (#2821), p-PI3K (#4228), PI3K (#4257), p-Akt (#9271), Akt (#9272), p-ERK (#4370), ERK (#9102), p-p38 (#9211), p38 (#9212) (all from Cell Signaling Technology, Beverly, MA, USA), and HSP-90 (ADI-SPA-836-F, Enzo Life Sciences, Farmingdale, NY). After a brief washing, membranes were incubated for one hour with either horseradish peroxidase-conjugated goat anti-mouse IgG (ADI-SAB-100) or goat anti-rabbit IgG (ADI-SAB-300, Enzo Life Sciences) at room temperature. Antibody signals were detected using a Las-4000 imager (GE Healthcare Life Science, Pittsburgh, PA, USA).

### Enzymatic cycling assay for quantitative NAD^+^ measurement

For NAD^+^ extraction, mast cells were washed with PBS, quenched with 180 μL of 0.6 M HClO_4_, and neutralized by the addition of 60 μL of 2 M KHCO_3_. For serum analysis of NAD^+^, 45 μL of serum were resuspended and neutralized in 45 µL of 0.6 M HClO_4_ and 30 μL of 2 M KHCO_3_, respectively. After centrifugation at 15,000 *g* for 10 min, the amount of NAD^+^ in the supernatant was measured by a cycling assay using a process described elsewhere 15. Briefly, the assay solution (2% ethanol, 100 μg/mL alcohol dehydrogenase, 10 μM resazurin, 10 μg/mL diaphorase, 10 μM flavin mononucleotide, 0.1 mg/mL bovine serum albumin, and 100 mM Na_2_HPO_3_ buffer, pH 7.0) was added to each sample and fluorescence changes were measured in a multiwell plate reader (with excitation at 544 nm and emission at 590 nm). After centrifugation, the pellets were re-dissolved in 0.4 M NaOH and quantified by Bradford assay (BIO-RAD, Hercules, CA, USA).

### Statistical analysis

Data are expressed as mean ± standard deviation of the mean (SD). All *in vitro* studies were performed in triplicate and repeated three times. All *in vivo* studies were performed with at least eight mice in each group. Statistical comparisons among multiple groups were made using one-way analysis of variance followed by Tukey's *post hoc* analysis. The significance of differences between two groups was determined using Student's unpaired *t*-test. A *p* value of less than 0.05 was considered significant. All analyses were performed using GraphPad Prism 9.3 (San Diego, CA, USA).

## Results

### NMN and NR dampen FcεRI-mediated degranulation in BMMCs and hCBMCs

**Figure 1:**
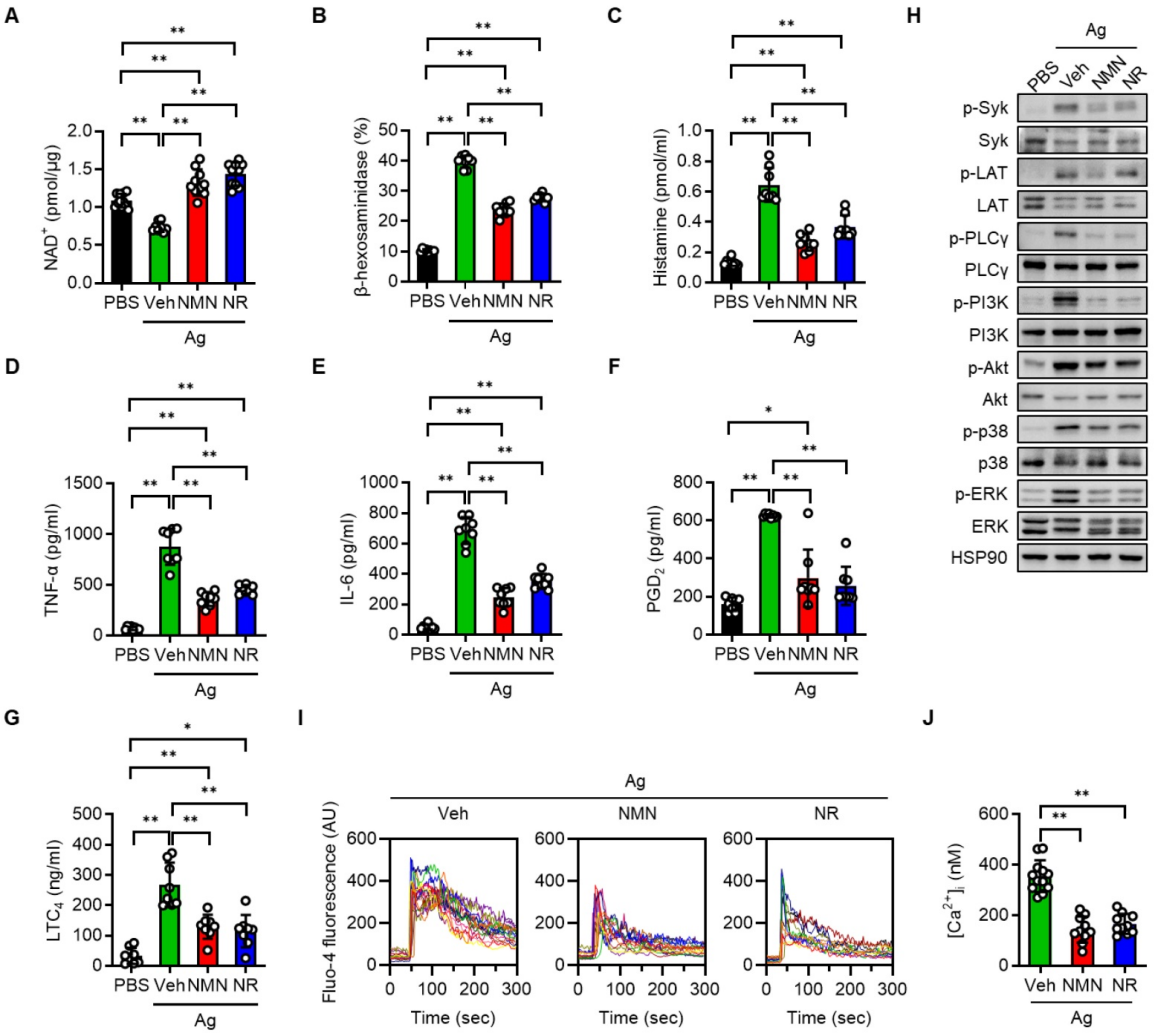
** NMN and NR suppress Ag-stimulated degranulation in BMMCs.** BMMCs (1 × 10^6^/mL) derived from C57BL/6J mice were sensitized overnight with 100 ng/mL anti-DNP-IgE and then treated with PBS (vehicle), 5 mM NMN, or 5 mM NR for 1 h. BMMCs were then stimulated with 100 ng/mL DNP-HSA (Ag). Degranulation was assessed by measurement of β-hexosaminidase, histamine, LTC_4_, and PGD_2_ at 30 min, and of IL-6 and TNF-α at 6 h post stimulation. (**A-F**) Intracellular NAD^+^ levels (n = 8-11), extracellular levels of β-hexosaminidase (n = 8), histamine (n = 8), TNF-α (n = 8), IL-6 (n = 8), PGD_2_ (n = 8) and LTC_4_ (n = 8) were analyzed. (**H**) After stimulation with Ag, cell lysates were prepared from BMMCs and used for the Western blot analysis. HSP90 was blotted as a loading control. Representative images are shown from three independent experiments. (**I**) IgE sensitized BMMCs were labeled with Fluo-4 and then stimulated with Ag for indicated times. Representative fluorescence emission spectral scans of intracellular calcium influx. (**J**) Quantification of intracellular Ca^2+^ concentrations [Ca^2+^]_i_ at 60 s after Ag stimulation (n = 10-12). Values are mean ± SD. ^*^p < 0.05 and ^**^p < 0.01. Ctrl, control; Veh, vehicle; NMN, nicotinamide mononucleotide; NR, nicotinamide riboside; PGD_2_, prostaglandin D_2_; LTC_4_, leukotriene C_4_

To investigate the protective function of NAD^+^ precursors on mast cell activation, we first set up the BMMCs, which were derived from normal C57BL/6J mice. Upon serial stimulation first with anti-DNP-IgE and then with DNP-HSA (Ag), we observed a significant decline of intracellular NAD^+^ pools in the BMMCs (Figure 1A). In contrast, pretreating the BMMCs with either NMN or NR prior to Ag stimulation led to an increase of intracellular NAD^+^ levels to control group levels. Notably, NMN or NR treatment did not result in a significant change in extracellular NAD^+^ levels (data not shown). In order to assess the impact of NAD^+^ boosting on the FcεRI-mediated degranulation response in the BMMCs, we measured the release of β-hexosaminidase and histamine into culture supernatants. As shown in Figure 1B-C, Ag-stimulated release of β-hexosaminidase and histamine was significantly suppressed by both NMN and NR treatment. A dose-respone experiment revealed that NMN and NR suppressed degranulation in BMMCs in a concentration-dependent manner within a range of 1 to 5 mM (Figure S1). Additional Ag-stimulated mediators that contributed to allergic inflammation, such as proinflammatory cytokines (TNF-α and IL-6) and arachidonic acid metabolites (PGD_2_ and LTC_4_), were also significantly suppressed by either NMN or NR (Figure 1D-G). To identify the responsible downstream signals of FcεRI, we stimulated BMMCs with Ag for 20 min in the presence of NMN or NR. The degree of phosphorylation of Syk, LAT, PLCγ, PI3K-Akt, p38 MAPK, and ERK was noticeably increased after Ag stimulation, while these changes were significantly suppressed in both NMN and NR-treated BMMCs (Figure 1H and S2). Because IgE-mediated degranulation is calcium dependent, we further analyzed the effects of NAD^+^ precursors on calcium levels using a fluorescent calcium indicator (Fluo-4 AM). As shown in Figure 1I-J, a robust increase in calcium oscillations and intracellular calcium concentrations was observed immediately upon Ag stimulation in vehicle-treated BMMCs, while NMN- or NR-treated BMMCs displayed a marked suppression of these changes. The concentrations of NMN and NR used in these experiments did not affect the viability of the BMMCs (data not shown).

To ascertain whether the inhibitory effects of NMN and NR on IgE-mediated BMMCs activation were a consequence of altered mast cell development, BMMCs from C57BL/6J mice were cultured in the presence of NMN or NR, and the maturation of the BMMCs was monitored. As shown in Figure S3A-B, cell surface expression of CD117 and FcεRI was significantly suppressed following both NMN and NR treatment. These results suggest that mast cell differentiation as well as mast cell degranulation is the target of NMN and NR.

**Figure 2:**
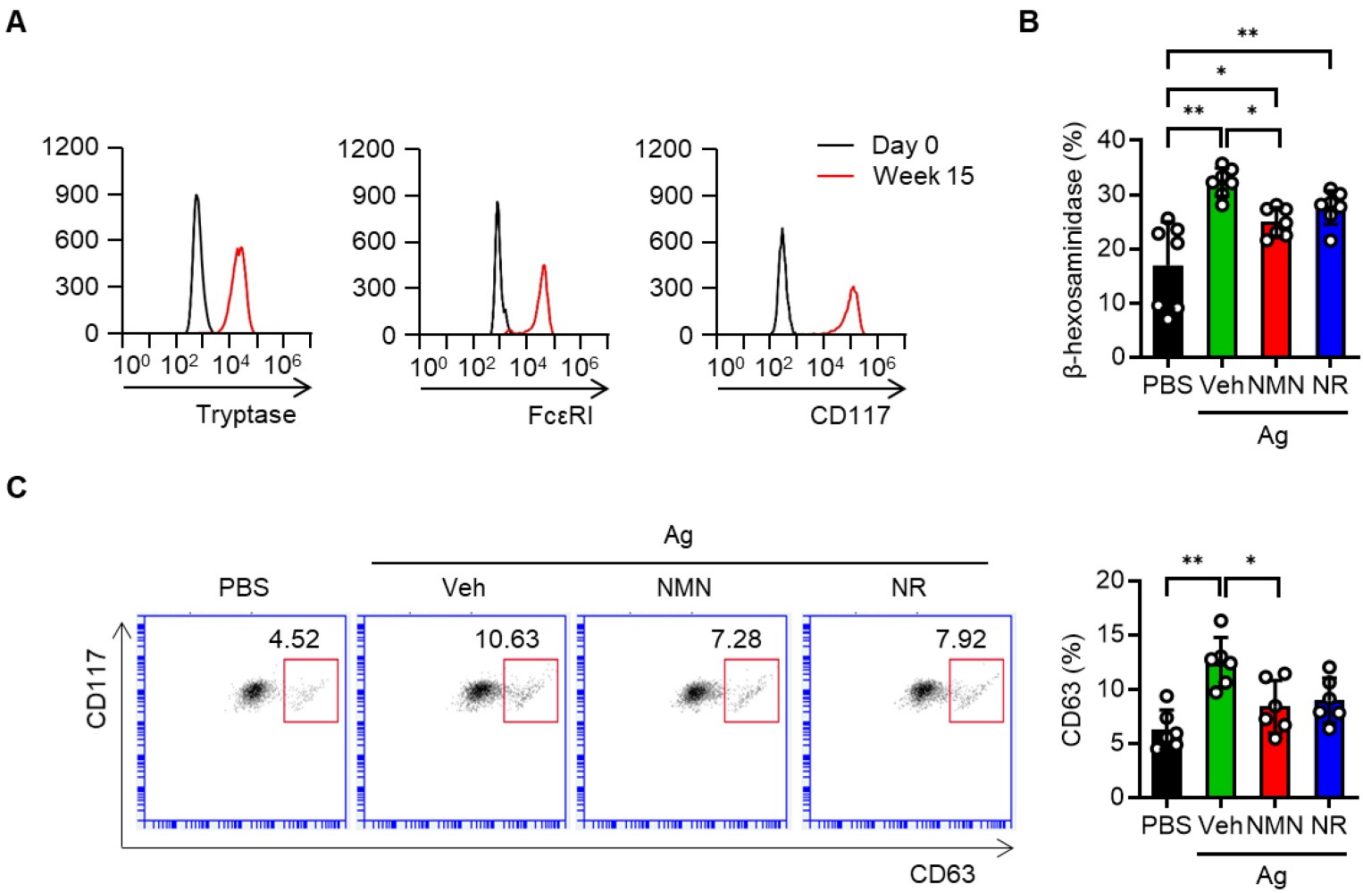
** NMN and NR suppress IgE/Ag-stimulated degranulation in human cord blood derived mast cells (hCBMCs).** (**A**) Flow cytometric analysis of tryptase, FcεRI and CD117 in hCBMCs. (**B, C**) After sensitization and stimulation with 100 ng myeloma IgE/100 ng anti-IgE (Ag), degranulation was determined by biochemical analysis of β-hexosaminidase releases (n = 7) and flow cytometric analyses of CD63 (n = 6). Values are mean ± SD. ^**^p < 0.01.

We next took advantage of hCBMCs to investigate whether the NAD^+^ precursors protected against human mast cell activation. Successful generation of the hCBMCs was confirmed by flow cytometric analysis of tryptase, FcεRI and CD117 (Figure 2A). These cells were sensitized with myeloma IgE and activated with anti-IgE in the presence or absence of NAD^+^ precursors. Similar to the results observed in murine BMMCs, the release of β-hexosaminidase and the surface expression of CD63 were suppressed by either NMN or NR pretreatment (Figure 2B-C). Together, these results indicate that NAD^+^ precursors are effective in suppressing Ag-stimulated FcεRI signaling and mast cell degranulation in murine and human mast cells *in vitro*.

### NMN and NR supplementation suppress PSA and PCA reactions

**Figure 3:**
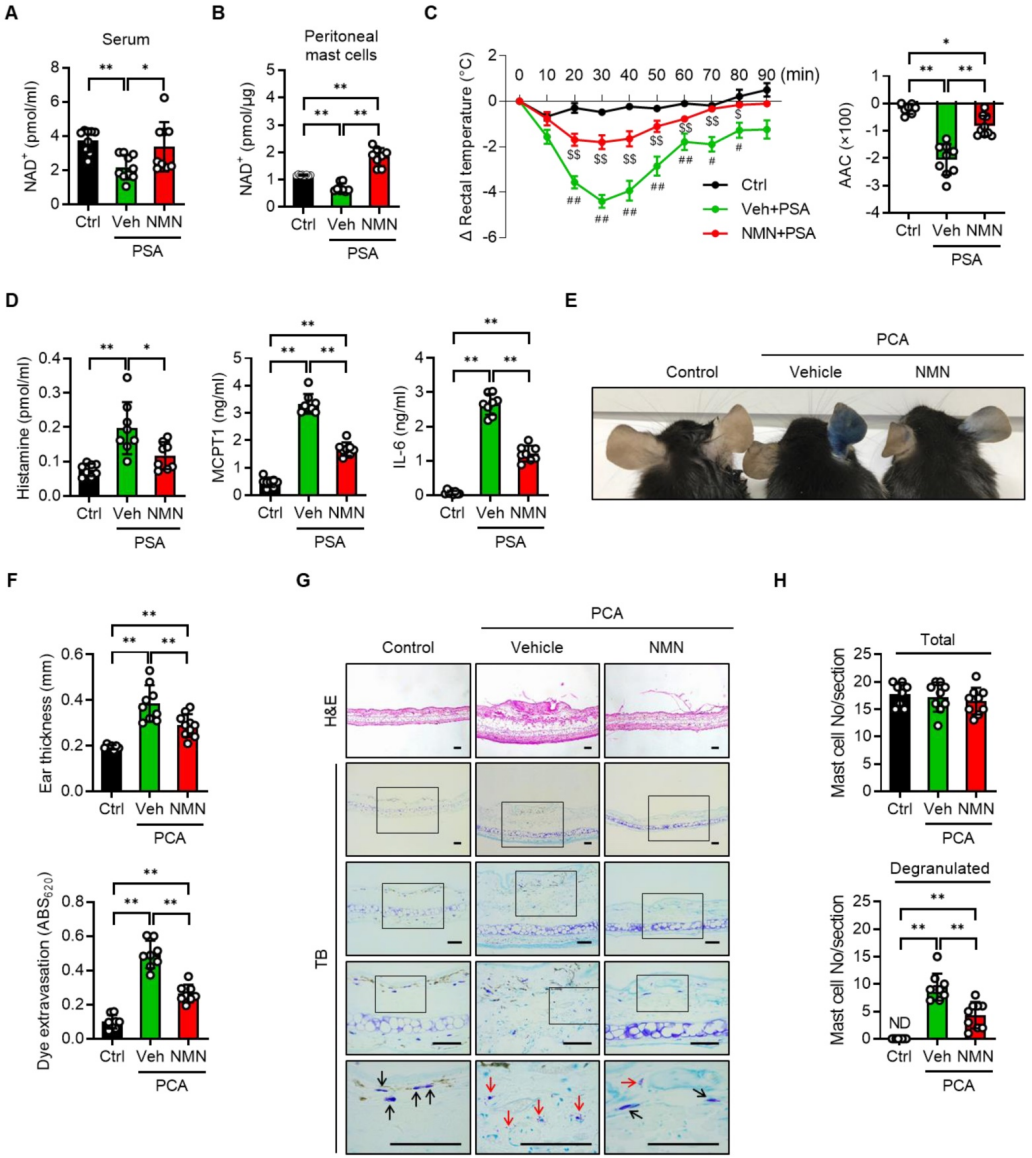
** NMN inhibits IgE-mediated anaphylactic response.** (**A-C**) C57BL/6J mice were sensitized with 10 μg anti-DNP-IgE and challenged with 100 μg DNP-HSA with or without intraperitoneal pretreatment of NMN (100 mg/kg). (**A**) Serum levels of NAD^+^ (n = 9-10), (**B**) NAD^+^ levels in peritoneal mast cells (n = 8), (**C**) changes in rectal temperature (n = 8-9), and (**D**) serum levels of histamine, MCPT1 and IL-6 (n = 8) were determined. Area above curve (AAC) was calculated as the area between the plotted curve and x-axis. (**E-H**) C57BL/6J mice were passively sensitized by intradermal injection of 1 μg anti-DNP-IgE into the ear. Subsequently, mice were challenged by intravenous injection of 100 μg DNP-HSA in PBS/Evans blue. (**E**) Representative ear images after PCA reaction and (**F**) ear swelling calculated by ear thickness (n = 8) and Evans blue dye extravasation (n = 8). (**G, H**) Representative H&E and toluidine blue (TB) staining of ear sections. Bars = 50 μm. Total and degranulated mast cells in ear skin section were counted (n = 8). Black and red arrows indicate non-degranulated and degranulated mast cells, respectively. Values are mean ± SD. ^*^p < 0.05 and ^**^p < 0.01; ^#^*p* < 0.05 and ^##^*p* < 0.01 vs. Ctrl;^ $^*p* < 0.05 and ^$$^*p* < 0.01 vs. Veh+PSA. Ctrl, control. Veh, vehicle; NMN, nicotinamide mononucleotide; MCPT1, mast cell protease 1; ND, not detected

We next investigated whether NMN and NR supplementation prevented a mast cell-mediated allergic response in mice with PSA or PCA. C57BL/6J mice were injected with either saline (vehicle) or NMN and then sensitized with IgE. Twenty-four hours after the third dose of NMN, we challenged mice with DNP-HSA to induce a PSA reaction and then monitored body temperature (Figure S4A). To determine whether the supplemented NMN was successfully converted to NAD^+^, we measured NAD^+^ levels in the mice serum and peritoneal mast cells. NAD^+^ levels were reduced in the PSA mice as compared to control mice, but were restored by NMN treatment (Figure 3A-B). In response to Ag challenge, C57BL/6J mice exhibited a significant and robust decrease in body temperature. However, treatment with 100 mg/kg NMN significantly attenuated the drop in body temperature to less than half of that of the vehicle-treated mice, and temperature remained elevated during the recovery phase (Figure 3C). Serum levels of histamine, MCPT1, and IL-6 at 100 min after the Ag challenge were consistently and significantly lower in NMN-treated mice than in the vehicle-treated mice (Figure 3D).

The therapeutic potential of NMN as an anaphylaxis treatment was confirmed in the PCA model by comparing changes in ear thickness and extravasation in mice. C57BL/6J mice were intradermally injected in the right ear with anti-DNP-IgE for sensitization and then injected intravenously with antigen (DNP-HSA) for challenge (Figure S4B). The mast cell degranulation-induced dye extravasation of each ear was measured. A marked dye extravasation in the right ear was observed in vehicle-treated PCA mice, while mice treated with NMN had reduced dye extravasation (Figure 3E and S5A-B). Accordingly, reductions in ear swelling and Evans blue dye extravasation were observed in NMN-treated mice (Figure 3F). A histologic examination of ear tissue revealed that there was less mast cell degranulation in NMN-treated mice, evidenced by the presence of numerous toluidine blue positive granules outside the mast cell cytoplasm in the vehicle-treated group (Figure 3G-H). Interestingly, the total number of mast cells in the ear induced by PCA was unaffected by NMN treatment.

**Figure 4:**
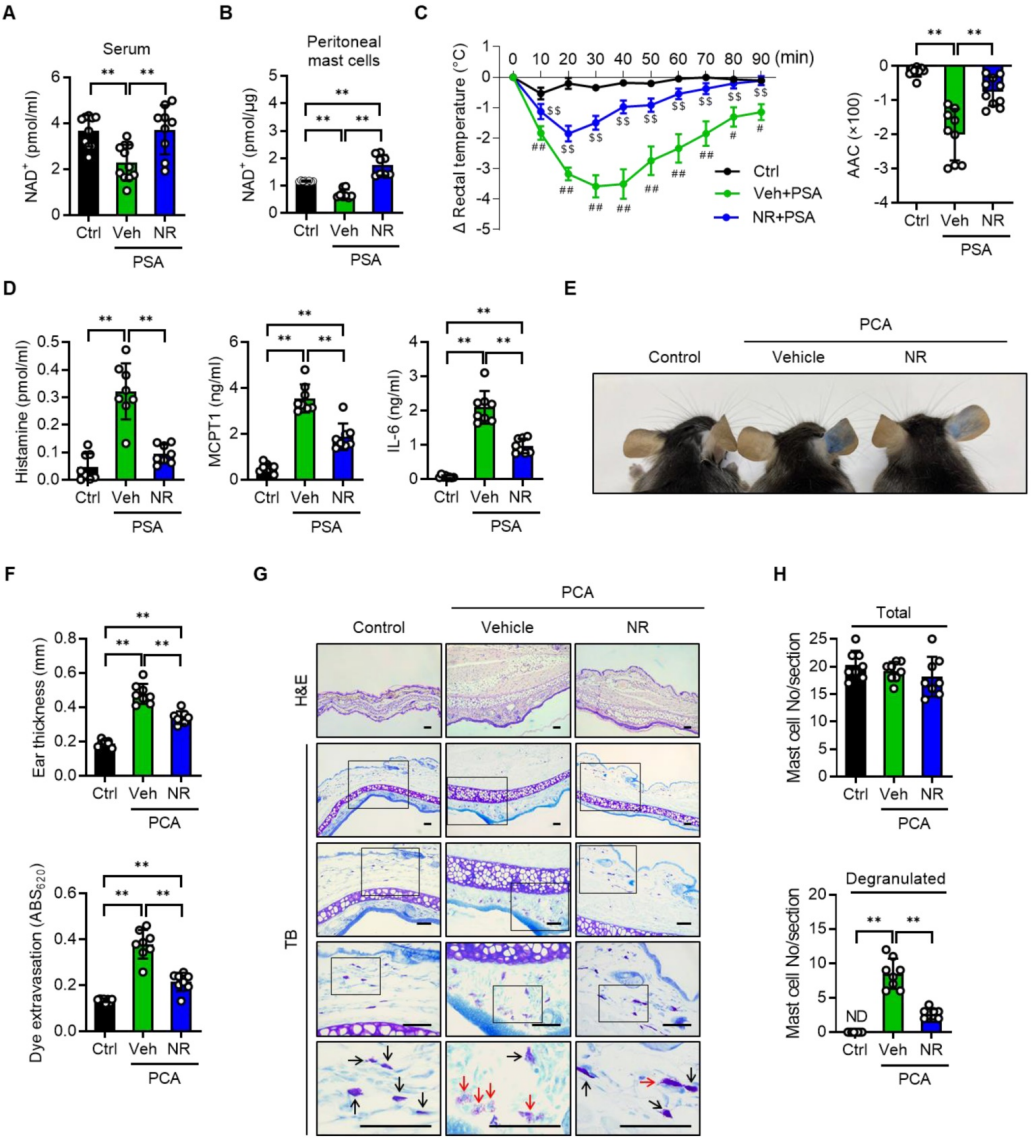
** NR protects IgE-mediated anaphylactic response.** All experimental procedures were same as described in Figure 3 legend except for the use of NR (150 mg/kg) instead of NMN. (**A**) Serum levels of NAD^+^ (n = 9-11), (**B**) NAD^+^ levels in peritoneal mast cells (n = 8), (**C**) changes in body temperature (n = 8), (**D**) serum levels of histamine, MCPT1 and IL-6 (n = 8), (**E**) representative ear images after PCA reaction, (**F**) ear swelling and Evans blue dye extravasation (n = 8), and (**G, H**) representative H&E and toluidine blue (TB) staining of ear sections and number of total- and degranulated-mast cells in ear skin section (n = 8). Bars = 50 μm. Black and red arrows indicate non-degranulated and degranulated mast cells, respectively. Values are mean ± SD. ^*^p < 0.05 and ^**^p < 0.01; ^#^*p* < 0.05 and ^##^*p* < 0.01 vs. Ctrl;^ $$^*p* < 0.01 vs. Veh+PSA. Ctrl, control; Veh, vehicle; AAC, area above curve; NR, nicotinamide riboside; MCPT1, mast cell protease 1; ND, not detected

Having elucidated NMN's beneficial role in attenuating anaphylactic reactions in mice, we also employed NR in mice with PSA or PCA. In agreement with the results obtained with NMN, NAD^+^ levels in serum and peritoneal mast cells, rectal temperature, serum levels of histamine, MCPT1 and IL-6 in the PSA model, as well as the cutaneous reactions including mast cell degranulation observed in the PCA model, suggested that NR treatment at 150 mg/kg also effectively suppressed anaphylactic reactions in mice (Figure 4A-H and S6A-B). The use of NMN and NR did not induce any signs of toxicity at the doses used (Table S1).

### NR supplementation's protective effect against anaphylaxis is partially abrogated in mast cell-specific Sirt6 KO mice

**Figure 5:**
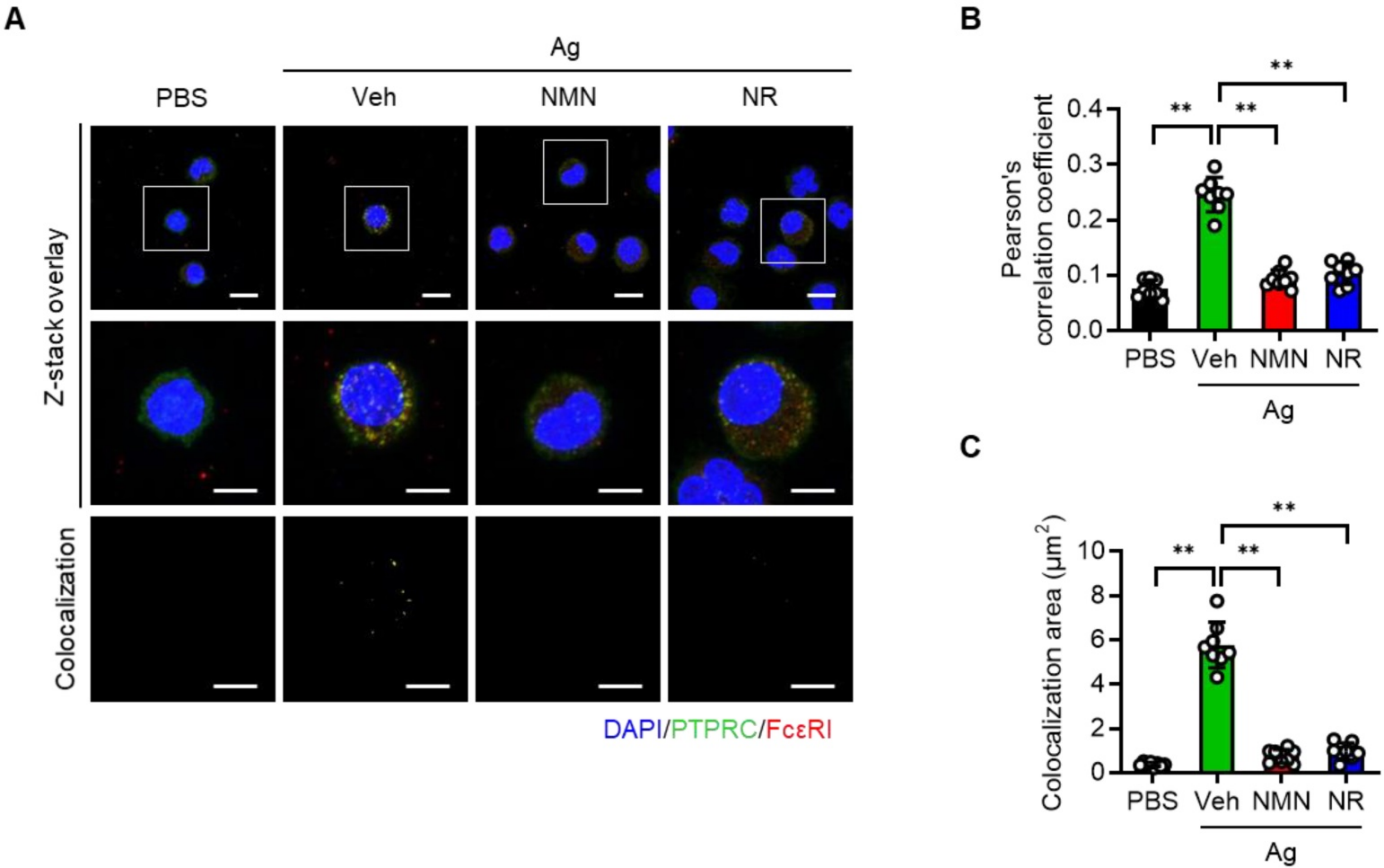
** NMN and NR suppress colocalization of PTPRC and FcεRI in BMMCs.** (**A**) Representative double immunofluorescence images of PTPRC and FcεRI in BMMCs. (**B, C**) Quantification of colocalization was carried out by calculating Pearson's correlation coefficient and by measuring colocalizaiton area (n = 8). Values are mean ± SD. ^**^p < 0.01.

Protein tyrosine phosphatase receptor type C (PTPRC, also known as CD45) is a transmembrane protein tyrosine phosphatase that activates FcεRI signaling 16. Recalling that NAD^+^ is a cofactor required for Sirt6 deacetylase function 17, and concluding that NAD^+^ precursors were found to significantly improve anaphylactic symptoms in mice (Figure 3 and 4), we hypothesized that NAD^+^ precursor supplementation would alleviate anaphylactic responses by enhancing Sirt6 activity. To test this hypothesis, we evaluated the effect of NMN and NR supplementation on anaphylactic responses in mast cell-specific *Sirt6* KO mice. As Sirt6 downregulates the expression of PTPRC 12, we first evaluated the effects of NMN and NR treatment on PTPRC expression in mast cells. As analyzed by confocal microscopy, Ag stimulation increased PTPRC expression as well as colocalization of FcεRI and PTPRC in BMMCs (Figure 5A-C). However these responses were markedly blunted by both NMN and NR treatment, implying the involvement of Sirt6 in the function of NMN and NR in mast cells.

**Figure 6:**
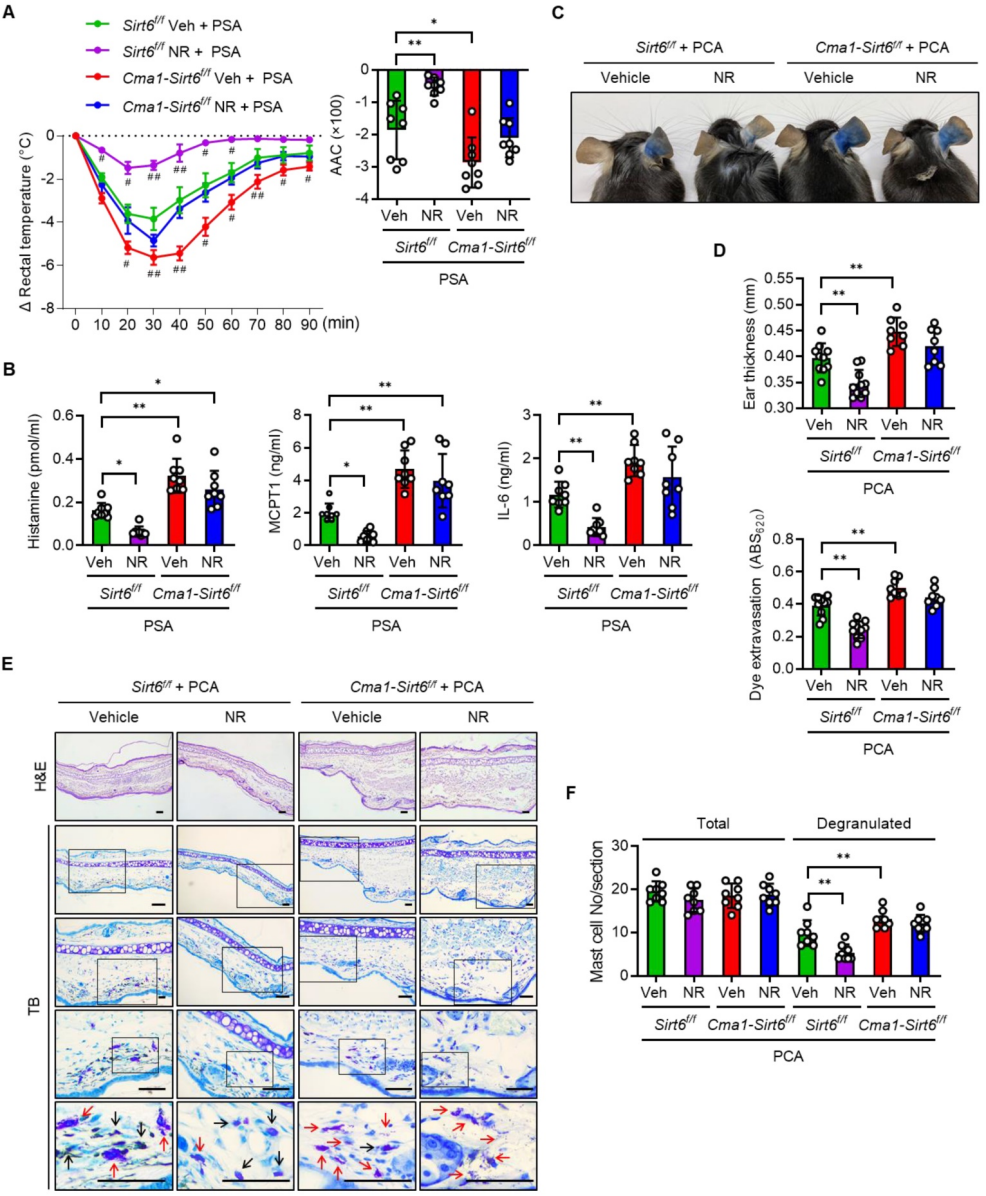
** NR does not protect IgE-mediated anaphylactic response in mast cell Sirt6 KO mice.** (**A-F**) All experimental procedures of PSA and PCA were identical to those described in the Figure 3 legend, except for the use of mast cell-specific* Sirt6* KO mice (*Cma1-Cre;Sirt6^flox/flox^*) and their wild-type littermates (*Sirt6^flox/flox^*). (**A**) Changes in rectal temperature (n = 8), (**B**) serum levels of histamine, MCPT1 and IL-6 (n = 8), (**C**) representative ear images after PCA reaction, (**D**) ear swelling and Evans blue dye extravasation (n = 8), and (**E, F**) representative H&E and toluidine blue (TB) staining of ear sections and number of total and degranulated mast cells in ear skin section (n = 8). Bars = 50 μm. Black and red arrows indicate non-degranulated and degranulated mast cells, respectively. Values are mean ± SD. ^*^p < 0.05 and ^**^p < 0.01; ^#^*p* < 0.05 and ^##^*p* < 0.01 vs. *Sirt6^f/f^* Veh+PSA. Veh, vehicle; NR, nicotinamide riboside; MCPT1, mast cell protease 1

We generated mast cell-specific *Sirt6* KO mice by crossing *Cma1-Cre* mice with floxed Sirt6 mice (Figure S7A-C), and investigated the effect of Sirt6 deficiency on anaphylaxis in mice with PSA or PCA. Mast cell-specific *Sirt6* KO mice exhibited the more severe body temperature drop upon Ag challenge as compared to their wild-type (WT) littermates, confirming the results of our previous study that uncovered the role of Sirt6 in preventing anaphylaxis 12 (Figure 6A). While NR treatment in WT mice almost completely prevented the decline of body temperature after Ag challenge, in *Sirt6* KO mice the NR exerted little protective effect, as reflected in the body temperature changes; NR-treated-*Sirt6* KO mice still showed a lower temperature than NR-treated WT mice. This result was further supported by data concerning serum levels of histamine and other cytokines (Figure 6B). Unsurprisingly, similar results were obtained in experiments that used the PCA model; ear swelling, extravasation, and mast cell degranulation were not significantly different between vehicle treated- and NR supplemented-*Sirt6* KO mice (Figure 6C-F). These results indicate that mast cell Sirt6 is indispensable for the attenuation of anaphylactic symptoms by NAD^+^ precursors.

## Discussion

In this study, we demonstrated for the first time that cellular NAD^+^ levels were diminished by anaphylaxis and that treatment with NAD^+^ precursors NMN and NR suppressed IgE-mediated mast cell degranulation and an anaphylactic response. Mechanistically, Sirt6 activation plays an essential role in the NAD^+^ precursors-mediated modification of mast cell degranulation. These results corroborate and extend our recent work regarding the therapeutic role of Sirt6 in protecting against Ag-stimulated mast cell degranulation and anaphylaxis.

While NAD^+^ physiology has long been studied, it is only relatively recently that more extensive research has revealed the therapeutic importance of NAD^+^ in many disease models. Systemic or cellular NAD^+^ levels decline with age in humans as well as in rodents 7, 18. NAD^+^ levels may also drop in response to pathologic conditions. Consequently, boosting NAD^+^ levels via NR administration represses disease progression in many aging- or inflammatory pathologies 19, 20 and has been shown to prolong yeast lifespans 21. We observed lower NAD^+^ levels in blood taken from anaphylactic mice, but found that levels could be restored by the intraperitoneal administration of NMN or NR. It is not clear what mechanisms contribute to the decline of NAD^+^ in those with anaphylactic conditions. One possible explanation is that the hyperactivation of CD38, an enzyme that cleaves NAD^+^ to generate nicotinamide and cyclic adenosine diphosphoribose (cADPR), may lead to a decrease in NAD^+^ content. Noah *et al*. 22, who showed that CD38 activation and consequent cADPR production are required for the passage of food allergens across the small intestine epithelium and induction of a food induced anaphylactic reaction, supports this idea.

The biological effect of NMN and NR administration has been studied in mice using a wide range of doses (from 100 mg/kg to 1000 mg/kg), as well as diverse treatment periods (single treatment to one-year treatment) and routes of administration (diet admixture or drinking water, oral gavage or intraperitoneal injection) 9. While the pharmacokinetic feature of NMN and NR remains unclear, both have been examined for their potential to increase NAD^+^ levels in plasma and other tissues regardless of route of treatment. NMN has been reported as rapidly appearing in plasma and peripheral tissues in mice within 15 min after one bolus intraperitoneal injection of NMN (500 mg/kg), leading to a marked increase in NAD^+^ in the liver after 1 h 23. The intraperitoneal injection of NR (50 mg/kg or 500 mg/kg) led to beneficial outcomes in mice 24. Taking account of these findings, as well as the fact that exogenous NMN functions in a transient manner 25, we decided to treat mice three times with NMN and NR at a maximal dose of 100 and 150 mg/kg respectively via intraperitoneal injection. Data from our dose response studies is shown in Figure S5 and S6, and reveals that 30 and 100 mg/kg of NMN and 75 and 150 mg/kg of NR effectively prevented anaphylactic responses in PCA models.

NAD^+^ plays an important role in several cellular processes, including ATP production, DNA repair, and histone deacetylation 5, 9, and NAD^+^ boosting may accordingly reduce toxic cellular injury under pathological conditions. Indeed, in the present study, we demonstrated that three doses of intraperitoneal NMN or NR induced a significant increase in NAD^+^ levels in the serum of mice, thereby preventing them from experiencing anaphylaxis. NR has been reported to be more efficient than NMN at restoring NAD^+^ levels in mice, possibly due to increased uptake 26. However, we identified no difference in the efficacy of NMR and NR in terms of their suppressive effect on anaphylactic symptoms or mast cell degranulation *in vitro*.

The molecular mechanism responsible for the effect of NAD^+^ precursors on mast cell degranulation likely involves protein deacetylase Sirt6. One recent study of ours suggested that allergen exposure increases Sirt6 expression in mast cells and that *Sirt6* deletion increases the release of histamine, lipid mediators, and cytokines involved in inflammation and hypersensitivity, resulting in anaphylaxis 12. In the current study, in the mast cell-specific *Sirt6* KO mice, responsiveness to Ag stimulation was augmented compared with wild-type anaphylactic mice, confirming that Sirt6 is a negative regulator of anaphylactic responses. More importantly, rescue from anaphylaxis by NR treatment was compromised in mast cell-specific *Sirt6* KO mice (*Cma1-Cre;Sirt6^flox/flox^*), suggesting that Sirt6 plays a critical but partial role as an effector molecule of NAD^+^ in preventing anaphylaxis. These results concurrently imply that the additional mediators besides Sirt6 may exist in the NAD^+^ function in anaphylaxis, including Sirt1, the activity of which is also controlled by the cellular NAD^+^/NADH ratio. Indeed, the most potent natural Sirt1 activator (resveratrol) 27 inhibits Ag-stimulated mast cell degranulation and alleviates allergic inflammation in mice 28. In contrast, mast cell-specific *Sirt1* KO mice (*Cma1-Cre;Sirt1^flox/flox^*) exhibit augmented passive cutaneous anaphylaxis (PCA) in response to an allergen challenge 29. These studies indicate that both Sirt1 and Sirt6 are critical to the suppression of Ag-stimulated mast cell degranulation. Additional studies that involve mice lacking other NAD^+^-consuming enzymes, such as poly(ADP-ribose) polymerase (PARP) or CD38, will be needed to determine the precise molecular mechanism that links NAD^+^ precursor supplementation to anaphylaxis.

While NAD^+^ precursor supplementation may exert a beneficial effect on anaphylaxis, potential complications exist. Some studies have reported that NAD^+^ boosting may increase the risk of cancers or accelerate tumor growth 30-32. In contrast, the oral administration of NAD^+^ precursors has been reported as reducing the size of precancerous skin lesions and preventing their progression to squamous cell carcinomas 33. Likewise, Sirt1, a primary mediator of NMN and NR's biological effects, may exert both pro- and anti-carcinogenic effects 34. One study has shown that NR supplementation decreases exercise performance in young rats 35, while the same treatment has been shown to improve exercise performance in a group of elderly (but not younger) human subjects 36. These studies suggest that more targeted and personalized approaches are needed to establish the efficacy and safety of NAD^+^ precursors as human therapeutics.

Sirt6 has been shown to regulate inflammation by deacetylating the non-histone or histone proteins of multiple immune/inflammatory cells. Specifically, Sirt6 suppresses Th2 cell differentiation by deacetylating GATA-3 37, enhances M2 macrophage polarization by activating the Akt pathway 38 while suppressing M1 macrophage polarization by inhibiting the STAT-3 pathway 39, accelerates eosinophil differentiation by stimulating GATA-1 transcriptional activity 40, and suppresses mast cell degranulation by reducing PTPRC expression on a plasma membrane without affecting its differentiation 12. These studies strongly suggest an anti-inflammatory role for Sirt6. Indeed, Sirt6 activation through either genetic overexpression or pharmacological activation is known to be effective against rheumatoid arthritis 41, allergic airway inflammation 37, non-alcoholic steatohepatitis 42, adipose tissue inflammation 43, and anaphylaxis 12. In this study, we observed that NMN and NR supplementation suppressed mast cell degranulation and anaphylaxis, and that Sirt6 was required to achieve these improvements. Read in light of the results obtained through earlier studies 12, 37, 41-43, our findings point to a potential use of NAD^+^ precursors in conjunction with Sirt6 activation as a therapeutic option for inflammatory disorders, including anaphylaxis.

## Supplementary Material

Supplementary figures and tables.

## Abbreviations

NAD^+^: nicotinamide adenine dinucleotide
NMN: nicotinamide mononucleotide
NR: nicotinamide riboside
PSA: passive systemic anaphylaxis
PCA: passive cutaneous anaphylaxis
BMMCs: bone marrow derived mast cells
hCBMCs: human cord blood derived mast cells
PTPRC: protein tyrosine phosphatase receptor type C
