# Supplementary material for: NAD+-boosting molecules suppress mast cell degranulation and anaphylactic responses in mice
Source: Theranostics. 2022 Apr 11;12(7):3316–28. doi: 10.7150/thno.69684 (PMC9065190; doi:10.7150/thno.69684)
Supplement: Supplementary file 1 — Supplementary figures and tables. [file thnov12p3316s1.pdf]

NAD$^+$-boosting molecules suppress mast cell degranulation and anaphylactic responses in mice

Hyun-Woo Kim[1,*], Ga-Hee Ryoo[2,*], Hyun-Young Jang[2,*], So-Young Rah[2], Dong Hyun Lee[3], Do-Kyun Kim[1], Eun Ju Bae[4,$], and Byung-Hyun Park[2,$]

[1]Korea Zoonosis Research Institute, Chonbuk National University, Iksan 54596, Republic of Korea

[2]Department of Biochemistry and Molecular Biology, Chonbuk National University Medical School, Jeonju 54896, Republic of Korea

[3]Department of Obstetrics and Gynecology, Chonbuk National University Medical School, Jeonju 54896, Republic of Korea

[4]College of Pharmacy, Chonbuk National University, Jeonju 54896, Republic of Korea

Contents

1. Supplementary table
2. Supplementary figures

1. Supplementary table

Table S1. Effects of intraperitoneal administration of NMN (100 mg/kg) and NR (150 mg/kg) on hematological and biochemical parameters in mice

	Hb (g/dL)	Hct (%)	WBC ($\times 10^3/\mu L$)	PLT ($\times 10^6/\mu L$)	AST (IU/L)	ALT (IU/L)	BUN (mg/dL)	Cr (mg/dL)
Vehicle	12.6±0.2	49±1	3.8±0.5	658±30	56±1	27±1	22±1	0.2±0.01
NMN	12.7±0.1	51±1	3.7±0.4	695±18	55±2	26±1	20±1	0.2±0.01
NR	12.8±0.3	50±2	3.8±0.3	665±38	54±3	27±1	21±1	0.2±0.01

Values are the mean ± SD (n=3). Hg, hemoglobin; Hct, hematocrit; WBC, white blood cell; PLT, platelet; AST, aspartate aminotransferase; ALT, alanine aminotransferase; BUN, blood urea nitrogen; Cr, creatinine

2. Supplementary Figures

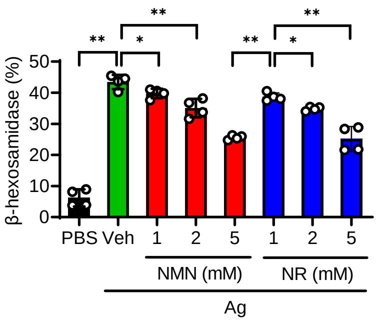

Figure S1. The dose-responsive effects of NMN and NR on Ag-stimulated degranulation in BMMCs. BMMCs (1 × 10^6/mL) derived from C57BL/6J mice were sensitized overnight with 100 ng/mL anti–DNP-IgE and then treated with indicated concentrations of NMN or NR. After 60 min of drug treatment, BMMCs were stimulated with 100 ng/mL DNP-HSA (Ag). The enzymatic activity of β-hexosaminidase released from the cells was measured at 30 min (n = 4). Values are mean ± SD. $^{*}p < 0.05$ and $^{**}p < 0.01$.

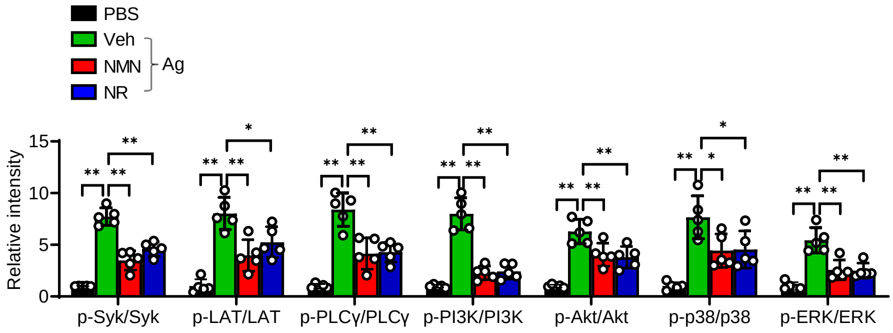

Figure S2. Suppression of FcεRI downstream signaling by NMN and NR. The band intensity shown in Figure 1H were quantified by densitometry (n = 5). Values are mean ± SD. $^{*}p < 0.05$ and $^{**}p < 0.01$.

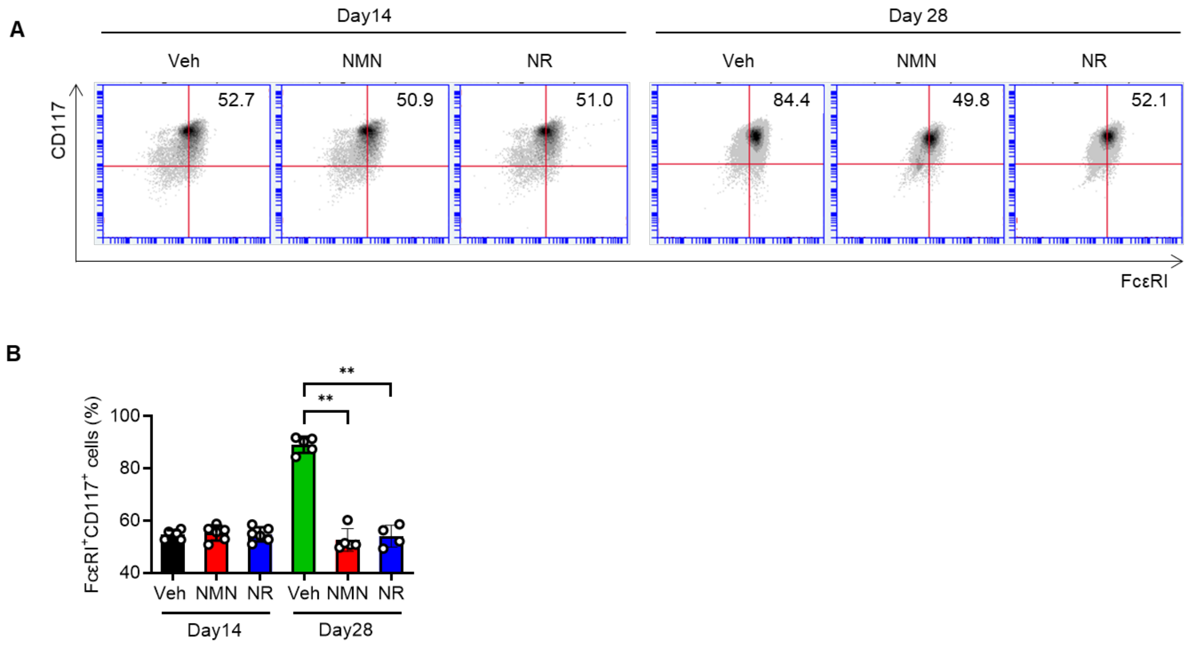

Figure S3. Effects of NMN and NR on BMMCs differentiation into mast cells. Bone marrow cells from C57BL/6J mice were induced to differentiate into mast cells with vehicle, 5 mM NMN or 5 mM NR. (**A**) Expression of FcεRI and c-Kit receptor in bone marrow–derived mast cells (BMMCs) was analyzed by flow cytometry. (**B**) Quantification of FcεRI^{+} and CD117^{+} cells (n = 4-6). Values are mean ± SD. $^{**}p < 0.01$.

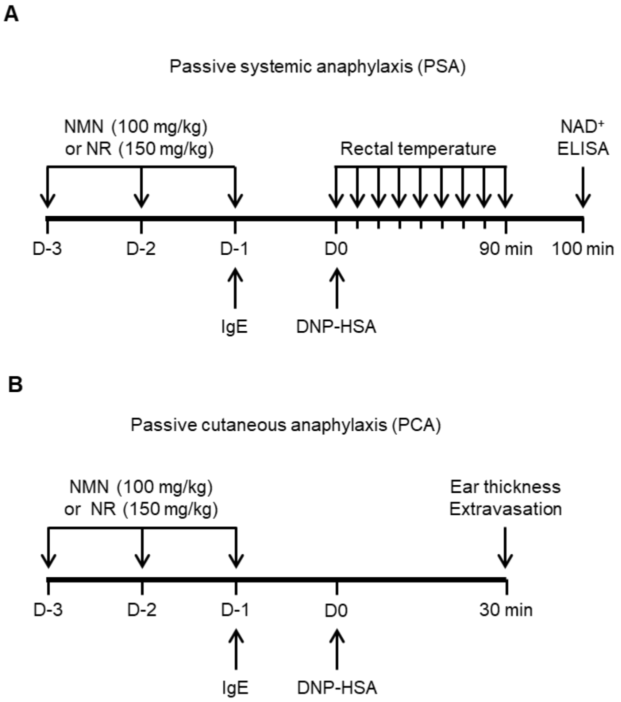

Figure S4. Schematic diagram of the experimental protocols. (**A**) NMN (100 mg/kg) or NR (150 mg/kg) was injected intraperitoneally three times to each mouse, once on days -3, -2, and -1. Mice were sensitized by intravenous injection with 10 µg of anti-DNP IgE in 100 µL PBS. On the following day, mice were intravenously challenged with 100 µg of DNP-HSA in 100 µL PBS. Rectal temperature and serum levels of NAD^+, histamine, MCPT1 and IL-6 were measured at the indicated time points. (**B**) NMN (100 mg/kg) or NR (150 mg/kg) was injected intraperitoneally three times to each mouse, once on days -3, -2, and -1. Mice were sensitized by intradermal injection with 1 µg of anti-DNP-IgE in 20 µL PBS in the right ear and an equal volume of saline in the left ear. After 24 h, mice were challenged intravenously with DNP-HSA (100 µg in saline containing 1% Evans blue). Thirty minutes after the challenge, the thickness of the ears was measured, and then mice were euthanized for the analyses of extravasation and mast cell degranulation.

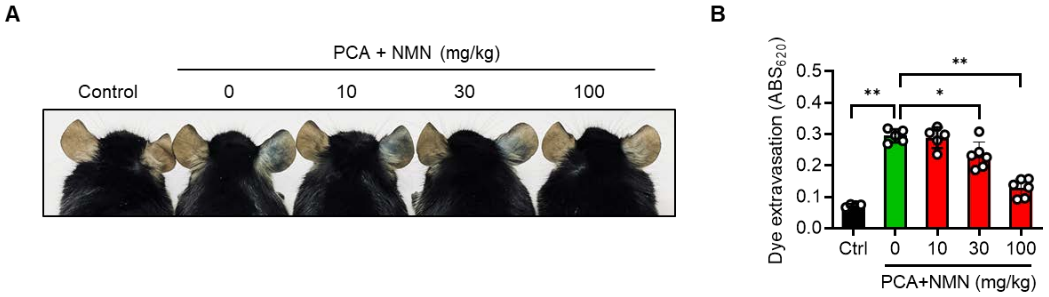

Figure S5. The dose-responsive effects of NMN on IgE-mediated anaphylactic responses. C57BL/6J mice were sensitized with 10 μg anti-DNP-IgE and challenged with 100 μg DNP-HSA with or without various doses of NMN. (**A**) Representative ear images after PCA reaction. (**B**) The Evans blue dye was extracted and quantified using a spectrophotometer (n = 4-6). Values are mean ± SD. $^{*}p < 0.05$ and $^{**}p < 0.01$.

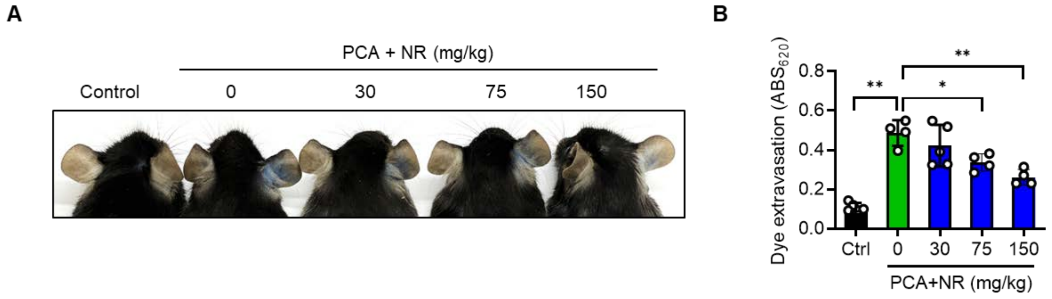

Figure S6. The dose-responsive effects of NR on IgE-mediated anaphylactic responses. C57BL/6J mice were sensitized with 10 μg anti-DNP-IgE and challenged with 100 μg DNP-HSA with or without various doses of NR. (**A**) Representative ear images after PCA reaction. (**B**) The Evans blue dye was extracted and quantified using a spectrophotometer (n = 4-5). Values are mean ± SD. $^{*}p < 0.05$ and $^{**}p < 0.01$.

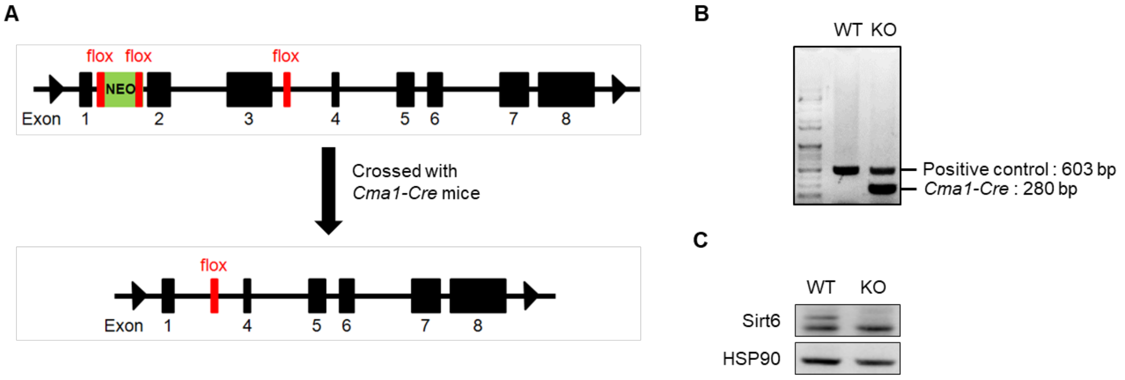

Figure S7. Generation of mast cell-specific *Sirt6* KO mice. (**A**) Schematic diagram illustrating mast cell-specific ablation of *Sirt6* using *Cma1-Cre*. (**B**) PCR analyses of genotyping from tail biopsies of *Cma1-Cre;Sirt6*$^{flox/flox}$ (KO) and their wild-type littermates (*Sirt6*$^{flox/flox}$, WT) mice. (**C**) Protein levels of Sirt6 in peritoneal mast cells of WT and *Sirt6* KO mice were compared by Western blotting.
